# MMP-Sensitive Macrophage-Targeted Coenzyme Q10 Nanomedicine for Rheumatoid Arthritis Treatment

**DOI:** 10.1021/acs.molpharmaceut.5c00742

**Published:** 2025-08-05

**Authors:** Aasma Akram, Nishat Ara, Prapanna Bhattarai, Muhammad Irfan, Lin Zhu

**Affiliations:** † Department of Pharmaceutical Sciences, Irma Lerma Rangel College of Pharmacy, 14736 Texas A&M University, College Station, Texas 77843, United States; ‡ Department of Pharmaceutics, Faculty of Pharmaceutical Sciences, 72594 Government College University Faisalabad, Faisalabad 38000, Pakistan

**Keywords:** nanomedicine, nanoparticles, macrophage targeting, rheumatoid arthritis, coenzyme Q10, inflammation

## Abstract

In this work, a novel nanoparticle (NP)-based formulation (i.e., nanomedicine) was developed to specifically deliver coenzyme Q10 (CoQ10) to macrophages for treating rheumatoid arthritis (RA). The NPs containing the matrix metalloproteinase (MMP)-responsive polymer, polyethylene glycol–peptide–phosphatidylethanolamine (PEG-pp-PE), and phosphatidylserine were designed, in which the NPs’ PEG “corona” decreased nonspecific interaction, and the overexpressed MMP-2/9 in the inflamed synovium triggered PEG deshielding and PS-mediated phagocytosis. The NPs were prepared by a thin-film hydration method, and their particle size, zeta potential, drug loading, and drug release were determined. CoQ10 could be loaded into the NPs with a drug loading of about 9.7% at the polymer/PS ratio of 50:50. The CoQ10-loaded NPs had a particle size of <200 nm and a zeta potential of ∼−40 mV and showed sustained drug release under simulated sink conditions at 37 °C. In the presence of MMPs, the NPs were efficiently taken up by macrophages (RAW264.7 cells) with cellular uptake 3.5-fold higher than that of the NPs in the absence of MMPs. More significantly, the NPs’ uptake in RAW264.7 cells was >13-fold higher than that in fibroblasts (NIH3T3 cells) in the presence of MMPs, whereas this difference was ∼2-fold in the absence of MMPs, indicating the NPs’ MMP-sensitive macrophage selectivity. In the LPS- and IFN-γ-stimulated macrophages or cell cocultures, the CoQ10-loaded NPs significantly inhibited the production of pro-inflammatory cytokines (TNF-α, IL-6, and IL-1β), while enhancing the production of the anti-inflammatory cytokine (IL-10) in response to MMP pretreatment. The CoQ10-loaded NPs also significantly inhibited macrophage activation, as evidenced by changes in cell morphology and alterations in macrophage activation-related markers, including CD80, CD86, MHCII, and CD206. The CoQ10-loaded NPs did not show significant cytotoxicity in the tested cells. Our results suggest that the developed MMP-sensitive macrophage-targeted NPs might work as a “smart” nanomedicine for RA treatments.

## Graphical abstract

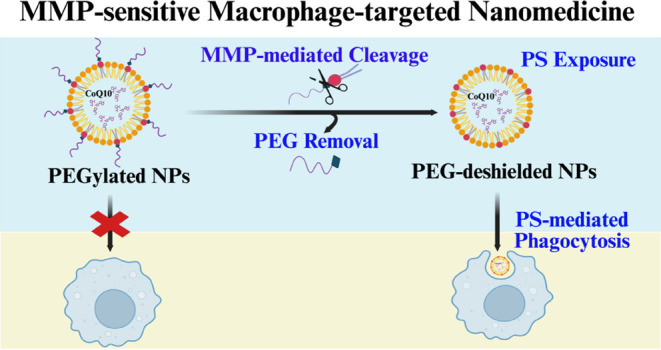

## 1 Introduction

Rheumatoid arthritis (RA) is an autoimmune disorder that affects around 1% of the population. Though major research has been carried out to study RA, its etiology remains vague.^1^ Pathophysiological studies have linked RA to inflammation, cartilage degradation, and bone erosion, during which macrophages are activated and have an appreciable role in RA progression.^2,3^ During RA, a large number of macrophages are recruited to the inflamed synovium and become activated to produce inflammatory mediators such as tumor necrosis factor-alpha (TNF-α), interleukin-6 (IL-6), interleukin-1 beta (IL-1β), prostaglandins, and reactive oxygen species (ROS), responsible for inflammation and bone damage.^4–6^ It is also an established fact that the presence of macrophages in the inflamed joint leads to an increased number of osteoclasts, resulting in bone resorption.^7,8^ Therefore, macrophages are considered to be an important therapeutic target for RA therapy.

The nonsteroidal anti-inflammatory drugs (NSAIDs), e.g., flurbiprofen, diclofenac, lornoxicam, diflunisal, etc., and disease-modifying antirheumatic drugs (DMARDs), e.g., hydroxychloroquine, azathioprine, methotrexate, etc., are currently used for RA treatment.^9,10^ However, serious adverse effects have been observed in patients with the long-term use of these drugs. Biologics, such as anti-TNF-α and anti-IL-6 antibodies, are also used to treat RA. Unfortunately, around 40% of RA patients do not respond to such antibody therapies^11,12^ and the treated patients may also be at higher risk of infections.^10,13^ Besides, these drugs are given systemically through the oral route or injection, which may cause nonspecific drug biodistribution and consequent toxicity or damage to healthy tissues and organs. Therefore, it is critical to develop safe and efficient medicines for RA treatment. A straightforward strategy is to use targeted drug delivery to improve the specificity of anti-RA drugs to macrophages in the inflamed tissues.^12^

Phosphatidylserine,^14^ an anionic cellular lipid, is found in the inner leaflet of the cell membrane, and its cellular location is maintained by amino phospholipid translocase in healthy cells. PS is genetically translocated from the plasma membrane’s inner side to its outer layer during cell apoptosis, and the PS externalization acts as an “eat me” signal to trigger phagocytosis.^15,16^ In addition to working as an immune modulator to suppress inflammation,^17–21^ PS has been used as a ligand in drug delivery systems to deliver drugs to areas with an abundance of phagocytic cells.^22–25^ However, PS is a “pan-specific” ligand and cannot differentiate between macrophages in disease sites and macrophages in healthy tissues. The disease specificity of PS-containing systems needs to be improved.

Matrix metalloproteinases (MMPs) are major extracellular enzymes that are zinc-dependent endopeptidases. MMPs are upregulated in inflammation-related diseases, such as cancer^26^ and RA.^27^ MMP2 and MMP9 have been found to be overexpressed in the joints of RA patients, contributing to inflammation and joint destruction.^28–31^ MMPs have been therapeutic targets for the development of MMP inhibitors. However, MMP inhibitors can be a double-edged sword in treating RA due to MMPs’ important roles of MMPs in both physiology and pathology.^32^ Alternatively, MMPs can work as local stimuli for on-demand “smart” drug delivery.^26^ So far, several MMP2/9-responsive delivery systems, such as liposomes^33,34^ and micelles,^35,36^ have been developed for targeted drug delivery to treat a variety of diseases, including cancer and inflammatory diseases such as RA.^27,37,38^ In our previous studies, the MMP-sensitive self-assembling copolymer PEG-pp-PE, which consists of an MMP-cleavable peptide (PP), polyethylene glycol (PEG), and phosphoethanolamine (PE), was synthesized for the preparation of MMP-responsive nanocarriers.^33,34,39,40^

Coenzyme Q10 (CoQ10) is an important hydrophobic endogenous antioxidant present in cell membranes.^41,42^ CoQ10 has also been reported to inhibit pro-inflammatory factors, including nuclear factor-kappa B (NF-κB), and promote anti-inflammatory factors, such as microRNA-146a (miR-146a) and nuclear factor erythroid 2-related factor 2 (Nrf2), resulting in the suppression of inflammation.^43,44^ Due to its poor aqueous solubility and low bioavailability, high doses of CoQ10 are usually needed, making drug formulation and administration difficult.

In this work, our goal was to develop an MMP2/9-sensitive drug delivery system for targeted delivery of CoQ10 to the activated macrophages in the inflamed synovium, to improve CoQ10’s anti-inflammatory effects and reduce its off-target adverse effects. We prepared the MMP-responsive PS-containing polymeric nanoparticles (NPs), a dual-targeting system, which could respond to the overexpressed MMPs in the inflammatory tissues to ensure site-specific drug delivery and mimic PS externalization for macrophage-specific drug uptake in the inflamed tissues (see the delivery strategy in Figure 1).

**Figure 1:**
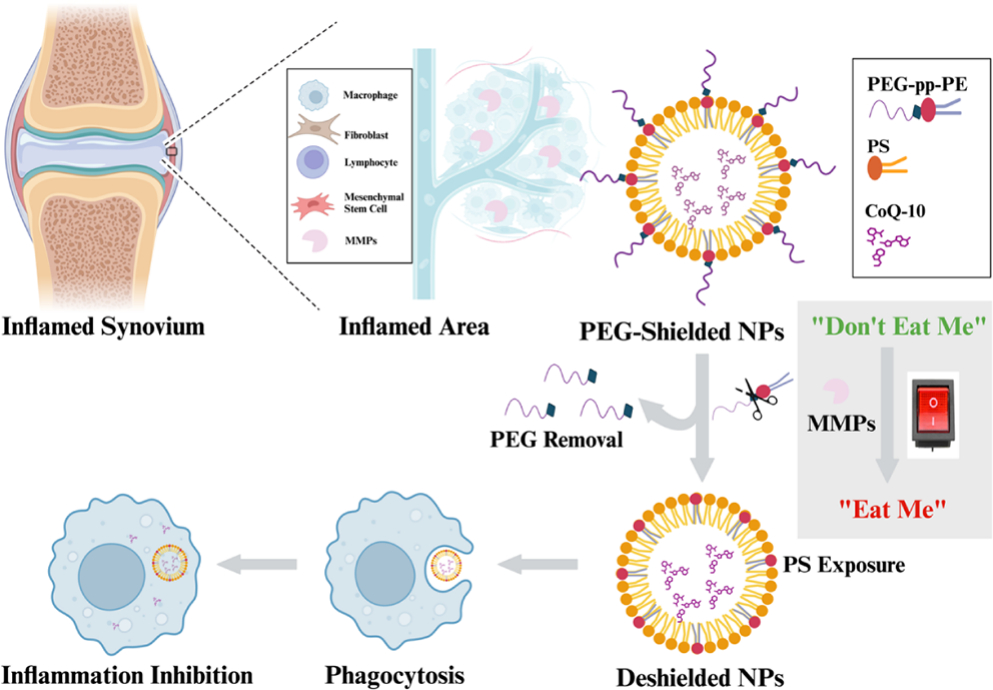
Schematic illustration of the CoQ10-loaded MMP-sensitive macrophage-targeted nanomedicine for RA treatment. In normal tissues with low levels of MMP2/9, the NPs’ polyethylene glycol (PEG) shields the PS and prevents nonspecific phagocytosis (to mimic “don’t eat me” signal). In the presence of overexpressed MMP2/9 in RA, the MMP-sensitive peptide linker (pp) is cleaved, leading to PEG deshielding and PS exposure on the surface of the NPs, which acts as an “eat me” signal for PS-mediated phagocytosis.

## 2 Materials and Methods

### 2.1 Materials and Cell Culture

The MMP-sensitive peptide (GPLGIAGQ) was synthesized by GenScript USA Inc. (Piscataway, NJ, USA). Methoxy polyethylene glycol (2,000 Da)-succinimidyl valerate (PEG2k-SVA) and 1,2-distearoyl-sn-glycero-3-phosphoethanolamine-N-[amino­(polyethylene glycol)-2000] (PEG2k-PE) were obtained from Laysan Bio, Inc. (Arab, AL, USA). 1,2-dipalmitoyl-sn-glycero-3-phospho-L-serine (sodium salt, DPPS), 1,2-dioleoyl-sn-glycero-3 phosphoethanolamine (DOPE), 1,2-dipalmitoyl-sn-glycero-3-phosphocholine (DPPC), and 1,2-dioleoyl-sn-glycero-3-phosphoethanolamine-N-(lissamine rhodamine B sulfonyl) (Rh-PE) were purchased from Avanti Polar Lipids, Inc. (Alabaster, AL, USA). Collagenase (Type IV), N-hydroxysuccinimide (NHS), and 1-(3-dimethylaminopropyl)-3-ethylcarbodiimide hydrochloride (EDC) were purchased from Sigma-Aldrich (St. Louis, MO, USA). Triethylamine (TEA) and dimethylformamide (DMF) were obtained from Thermo Fisher Scientific (Rockford, IL, USA). The glass-backed TLC plates coated with silica gel 60 F254 were purchased from EMD Biosciences (La Jolla, CA, USA). CoQ10 was purchased from Chem-Impex International (Wood Dale, IL, USA). Dulbecco’s Modified Eagle’s Medium (DMEM), heat-inactivated fetal bovine serum (FBS), and Penicillin-Streptomycin were purchased from Corning Life Sciences (Tewksbury, MA, USA). All other ingredients were of analytical grade.

Mouse macrophages (RAW 264.7) and fibroblasts (NIH 3T3) were obtained from the American Type Culture Collection (ATCC) (Manassas, VA, USA). Both cell types were maintained in complete growth media (DMEM supplemented with 10% FBS and 1% Penicillin-Streptomycin) at 37 °C in a humidified atmosphere with 5% CO_2_.

### 2.2 Polymer Synthesis and Nanoparticle Preparation

PEG-pp-PE was synthesized in accordance with our previously reported method.^33,39^ The polymeric NPs were prepared via self-assembly by using the thin-film hydration method. In this study, three types of NPs were prepared, including **STNPs** (*PEG-pp-PE/PS*), the MMP- **S** ensitive macrophage **T** argeting **NPs** ; **SNNPs** (*PEG-pp-PE/PC*), the MMP- **S** ensitive macrophage **N** ontargeting **NPs** ; and **NTNPs** (*PEG-PE/PS*), the **N** onsensitive macrophage **T** argeting **NPs** . For STNPs, three formulations with different PEG-pp-PE/PS ratios (75/25, 50/50, or 25/75 wt %) were prepared for formulation optimization.

Here, STNPs were used as an example to explain the preparation procedure. Briefly, PEG-pp-PE and DPPS were dissolved in a methanol and chloroform mixture and dried to form a thin lipid-polymer film by solvent evaporation. Then, the thin film was hydrated with PBS by vortexing and mild probe sonication. To prepare fluorescent dye- or drug-loaded NPs, Rh-PE (0.5 wt %) or CoQ10 dissolved in methanol was added to the PEG-pp-PE and DPPS mixture, followed by the thin-film hydration method.

### 2.3 Drug Loading and Encapsulation Efficiency

The drug loading and encapsulation of STNPs were measured. A 1 μg portion of the STNP formulation (PEG-pp-PE and PS) and 0.1 mg of CoQ10 were dissolved in an organic solvent and dried to form a drug-containing thin film, followed by probe sonication. The STNPs were centrifuged at 14,000 rpm for 15 min to remove unencapsulated drugs. The supernatant with the drug-loaded NPs was collected. To measure the CoQ10 content, methanol was added to the NPs to extract encapsulated drugs, followed by UV absorbance measurement at the λ_max_ of 275 nm with a microplate reader (Tecan). The following formulas were used to determine the drug loading and encapsulation efficiency.

$$\mathrm{Drug loading}\;(\%)=\frac{\mathrm{Weight of encapsulated drugs}}{\mathrm{Weight of drug‐loaded nanoparticles}}\times 100\%$$

$$\mathrm{Encapsulation efficiency}\;(\%)=\frac{\mathrm{Weight of encapsulated drugs}}{\mathrm{Total drugs}}\times 100\%$$

### 2.4 Particle Size and Zeta (ζ) Potential Measurement

For the measurement, the NPs were prepared in pH 7.4 PBS. The particle size, size distribution, and polydispersity index (PDI) of the NPs were evaluated by using Dynamic Light Scattering (DLS) with a NanoBrook Omni Particle Analyzer (Brookhaven Instruments Corporation). Measurements were conducted at 25 °C in a 10 mm cuvette with detection at a 90° angle. The ζ potential was determined by Phase Analysis Light Scattering (PALS) by using the same machine. Moreover, the change in NPs’ particle sizes was monitored at different pH levels and temperatures to estimate NP stability.

### 2.5 *In Vitro* Drug Release Study

To test the release of CoQ10 from STNPs, the dialysis method was used. Briefly, the drug-loaded NPs were placed in a dialysis bag (MWCO: 12–14 kDa). The dialysis bag was immersed in pH 7.4 Tween 80 (0.5%, w/v)-containing PBS to mimic the “sink” condition and subjected to stirring at 37 °C for up to 48 h. Samples were collected from the outer medium at prescheduled time points. The released drug was measured at the λ_max_ of 275 nm by a microplate reader (Tecan). To study MMP-sensitive drug release, the NPs were preincubated with 100 μg/mL collagenase IV in a pH 7.4 50 mM HEPES-buffered saline solution (150 mM NaCl and 10 mM CaCl_2_) at 37 °C for 4 h prior to the study of drug release.^33,34^ The difference factor (f1) and similarity factor (f_2_) were calculated to compare the drug release profiles of the free drugs and NP formulations using DDSolver software.^45–47^

### 2.6 PS Exposure/“Externalization” Analysis

To confirm the MMP sensitivity and PS exposure on the surface of the NPs, the FITC-labeled annexin V protein was used as a probe.^34^ Briefly, the samples of PS, PC, and PEG-pp-PE/PS (STNPs) were preincubated with 100 μg/mL collagenase IV at 37 °C for 4 h. Then, the samples were incubated with FITC-annexin V in the dark at room temperature for 15 min, followed by analysis using a BD Accouri C6 flow cytometer.

### 2.7 Cellular Uptake Study

To study cellular uptake, RAW 264.7 or NIH/3T3 cells were seeded in 96-well plates with 1 × 10^4^ cells/well and incubated overnight. To investigate the impact of MMPs on cellular uptake, the Rh-PE-loaded NPs were prepared and pretreated with 100 μg/mL collagenase IV at 37 °C in the dark for 4 h. The cells were treated with the NPs in serum-free DMEM at 37 °C for 1 h. Then, the cells were rinsed with PBS to eliminate uninternalized NPs and observed under a Nikon Eclipse Ti fluorescence microscope. To quantify the uptake, the cells were trypsinized, collected, and analyzed with a BD Accouri C6 flow cytometer. Viable cells were distinguished from dead cells and debris based on their forward versus side scatter characteristics.

### 2.8 Cytotoxicity Study

The cytotoxicity of the NP formulations was analyzed by the CellTiter-Blue Cell Viability Assay (Promega). Briefly, the cells (3 × 10^3^ cells/well) were seeded in 96-well plates and incubated overnight before the treatments. The formulations, including CoQ10, empty STNPs, and CoQ10-loaded STNPs, were incubated with the cells in complete growth media at 37 °C for 46 h. Then, 10 μL of CellTiter-Blue reagent was added to the cells and incubated for an additional 2 h. The fluorescence of the treated cells was determined at λ_ex_ of 560 nm and λ_em_ of 590 nm using a microplate reader (Tecan).

### 2.9 Determination of Cytokine Production

The cytokine production in the RAW 264.7 cells was determined by ELISA (eBioscience Inc.) according to the manufacturer’s manual. Briefly, the cells (1 × 10^5^ cells/well) were seeded in 12-well plates and incubated overnight before the treatments. In this experiment, five treatments were evaluated, including: (1) Untreated; (2) LPS (100 ng/mL) + IFN-γ (20 ng/mL) activation; (3) LPS (100 ng/mL) + IFN-γ (20 ng/mL) activation, then CoQ10; 4) LPS (100 ng/mL) + IFN-γ (20 ng/mL) activation, then CoQ10-loaded STNPs with MMP pretreatment; and (5) LPS (100 ng/mL) + IFN-γ (20 ng/mL) activation, then CoQ10-loaded STNPs without MMP pretreatment. After 24 h of activation, the cells were incubated with the formulations for an additional 24 h. Then, the cell medium was collected, followed by ELISA.

### 2.10 Macrophage Surface Marker Analysis

In this experiment, five treatment groups were evaluated, including: (1) Untreated; (2) LPS (100 ng/mL) + IFN-γ (20 ng/mL) activation; (3) LPS (100 ng/mL) + IFN-γ (20 ng/mL) activation, then CoQ10; 4) LPS (100 ng/mL) + IFN-γ (20 ng/mL) activation, then CoQ10-loaded STNPs with MMP pretreatment; and 5) LPS (100 ng/mL) + IFN-γ (20 ng/mL) activation, then CoQ10-loaded STNPs without MMP pretreatment. Briefly, the RAW 264.7 cells (1 × 10^5^ cells/well) were seeded in 12-well plates and incubated overnight. The cells were activated for 24 h, followed by incubation with the formulations for an additional 24 h. Then, the cells were trypsinized, collected, and incubated with FITC-labeled antibodies against macrophage surface markers for 30 min on ice, followed by flow cytometry on a BD Accouri C6 flow cytometer.

### 2.11 Inflammation Inhibition in Cell Co-Cultures

The macrophages (RAW 264.7) and fibroblasts (NIH/3T3) at 1 × 10^5^ cells/well (1:1 ratio) were seeded in 6-well plates and incubated overnight to generate the cell coculture. To mimic RA inflammation, the coculture was pretreated with LPS and IFN-γ. Briefly, five treatments were assessed in this model, including: (1) Untreated; (2) LPS (100 ng/mL) + IFN-γ (20 ng/mL) activation; (3) LPS (100 ng/mL) + IFN-γ (20 ng/mL) activation, then CoQ10; (4) LPS (100 ng/mL) + IFN-γ (20 ng/mL) activation, then CoQ10-loaded STNPs with MMP pretreatment; and (5) LPS (100 ng/mL) + IFN-γ (20 ng/mL) activation, then CoQ10-loaded STNPs without MMP pretreatment. After 24 h of treatment, the cytokines and cell surface markers were determined.

### 2.12 Macrophage Morphology

The impact of the CoQ10 nanomedicine on macrophages’ morphology was also examined. RAW 264.7 cells were seeded in a 35 mm dish with 14 mm glass bottom at a density of 1 × 10^5^ cells/well and incubated overnight before the experiments. The cells were preactivated with LPS (100ng/mL) + IFN-γ (20 ng/mL) for 24 h. Then, the cells were treated with CoQ10-loaded STNPs at 37 °C for 4, 8, or 12 h. The morphological changes in the cells were analyzed by using a Nikon Eclipse Ti fluorescence microscope.

### 2.13 Statistical Analysis

Three independent experiments were performed to obtain quantitative data. The data were expressed as mean ± standard deviation.^48^ For statistical analysis, the three formulations were analyzed using one-way ANOVA, while paired *t*-tests were performed to evaluate differences before and after drug loading within each formulation. Moreover, the STNPs with and without the MMP pretreatment were analyzed by unpaired *t*-test using GraphPad Prism (version 10.4.1) (San Diego, CA, USA). *­(*p* < 0.05), **­(*p* < 0.01), or ***­(*p* < 0.001) indicate a statistically significant difference.

## 3 Results and Discussion

CoQ10 is an endogenous ubiquinone that is mainly synthesized in the mitochondrial inner membrane and is present in most cell membranes.^49^ CoQ10 is a lipophilic molecule and plays a crucial role in cellular energy production and many other processes. Among its many functions, CoQ10 shows its anti-inflammatory effects mainly by inhibiting inflammation-related genes, e.g., *NF-κB*, and inducing genes that suppress inflammation, e.g., *miR-146-a* and *Nrf2*.^43,44^ CoQ10 also acts as a potent antioxidant, preventing cells from oxidative damage caused by free radicals^50^ to prevent or ameliorate inflammation. However, due to its high lipophilicity, CoQ10 shows poor water solubility and low bioavailability, even though it can easily diffuse through cell membranes.^44^ This poses a significant challenge in CoQ10’s formulation development and clinical applications.^51,52^ To date , numerous efforts have been made to increase CoQ10’ solubility and bioavailability.

In our prior research, we synthesized several MMP2/9-sensitive copolymers and conjugates and demonstrated their potential in targeted drug delivery and controlled drug release.^33,35,39,53,54^ Among them, the MMP2/9-sensitive self-assembling copolymers, PEG-pp-PE, have been used in the preparation of different types of nanomedicine with improved tumor specificity and on-demand drug release.^33,34,39,40^ In this work, using the MMP-sensitive copolymer (PEG-pp-PE) along with the “eat me” ligand PS, we prepared an inflammation- and macrophage-dual-targeting nanocarrier for CoQ10 delivery and RA treatment. In this design, MMP2/9 works as a “switch” to control the transition from “don’t eat me” (PEG shield) to “eat me” (PS exposure) of the CoQ10-loaded NPs (nanomedicine). In inflamed tissues, the upregulated MMPs cleave PEG-pp-PE to remove the PEG and expose the PS on the surface of NPs for macrophage-targeted phagocytosis (Figure 1).

### 3.1 Nanoparticle Preparation and Drug Loading

In previous studies, like commercially available PEG-PE polymers, the MMP-sensitive PEG-pp-PE was able to self-assemble and form polymeric micelles with a lipid core capable of encapsulating lipophilic drugs.^39,55^ In this study, the lipid^14^ and PEG-pp-PE together would self-assemble into an NP under mild sonication to form a similar “core-shell” nanostructure with a lipid (PE + PS) core and a PEG shell Figure 2.

**Figure 2:**
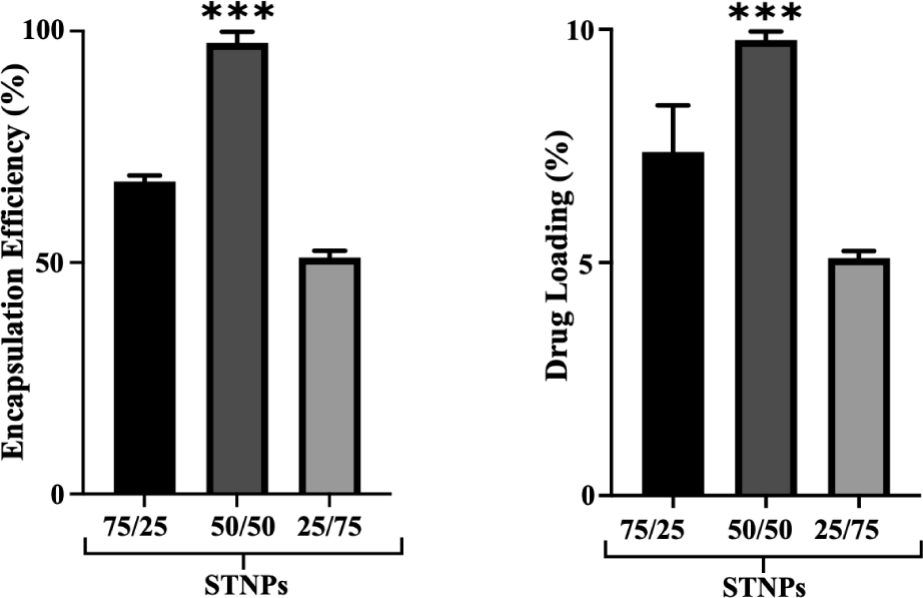
Encapsulation efficiency and drug loading of the PEG-pp-PE/PS (75/25, 50/50, and 25/75 wt %) self-assembled NPs (STNPs) (mean ± SD, *n* = 3). STNP (50/50) was compared with STNP (75/25) and STNP (25/75). ***p < 0.001, statistically significant.

Due to its high lipophilicity, CoQ10 is poorly water-soluble. The NP formulations with three different PEG-pp-PE/PS ratios were prepared to examine their drug-loading capability. We found that the lipophilic CoQ10 was readily incorporated into the lipid core of all PEG-pp-PE/PS NPs during NP formation (self-assembly), probably through hydrophobic interactions.^56^ Among the formulations, the STNPs (PEG-pp-PE/PS, 50/50 wt %) showed the highest encapsulation of 97.46 ± 2.37% and drug loading of 9.74 ± 0.23%. This might be due to the excellent self-assembly of PEG-pp-PE and PS at the ratio of 50/50 and the increased volume of the lipid core (both PE and PS) of the NPs.

### 3.2 Particle Size and Zeta Potential

The particle size and zeta potential of nanoparticles are crucial for maintaining colloidal stability by preventing aggregation through electrostatic repulsion among particles. For drug delivery, the nanoranged size and proper surface charge can not only help prevent NP aggregation but also reduce the likelihood of rapid clearance and prolong NPs’ *in vivo* half-life.^57^

The NP’s particle size was evaluated by DLS (Figure 3). The mean particle size of the empty NPs ranged from 146.83 ± 3.21 nm to 178.00 ± 5.71 nm. The CoQ10 loading changed the particle sizes of the NPs; however, the particle sizes of the drug-loaded NPs remained within the range of 100–200 nm, which is considered appropriate for drug delivery.^58–60^

**Figure 3:**
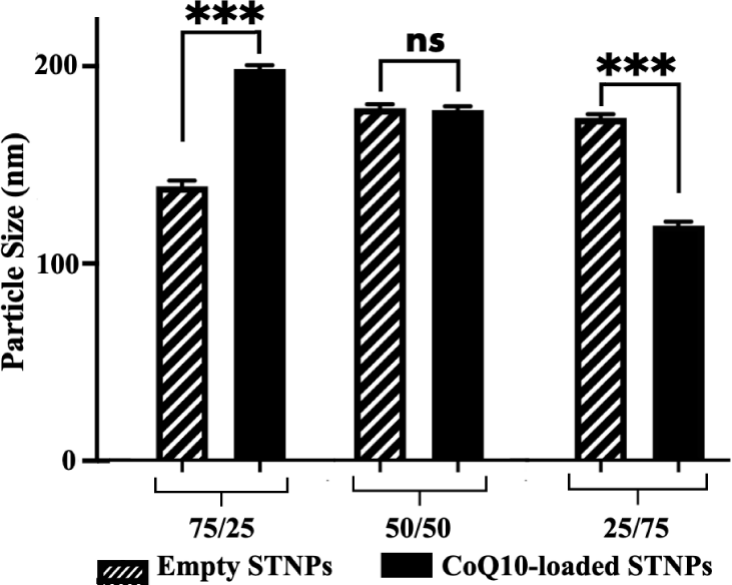
Mean particle size of self-assembled PEG-pp-PE/PS NPs (STNPs) at pH 7.4 with or without loading of CoQ10 (mean ± SD, *n* = 3). ns, not statistically significant; ***p < 0.001, statistically significant.

Among the STNP formulations, STNPs (50/50) with or without CoQ10 showed a similar mean particle size (Figure 3) and size distribution, which might indicate their stable nanostructure (Figure 4A). The PDI data for empty and CoQ10-loaded NPs ranged from 0.089 ± 0.024 to 0.369 ± 0.086 (Figure 4B), indicating a homogeneous distribution of NPs with respect to their particle size and stability.^14,61,62^ Among the formulations, STNPs (50/50) showed the lowest PDI. Also, considering the highest drug loading and encapsulation of STNPs (50/50) (Figure 2), the formulation STNPs (50/50) was selected for further experiments, although their particle size with the drug (175.94 ± 3.43 nm) was slightly larger than that of other formulations.

**Figure 4:**
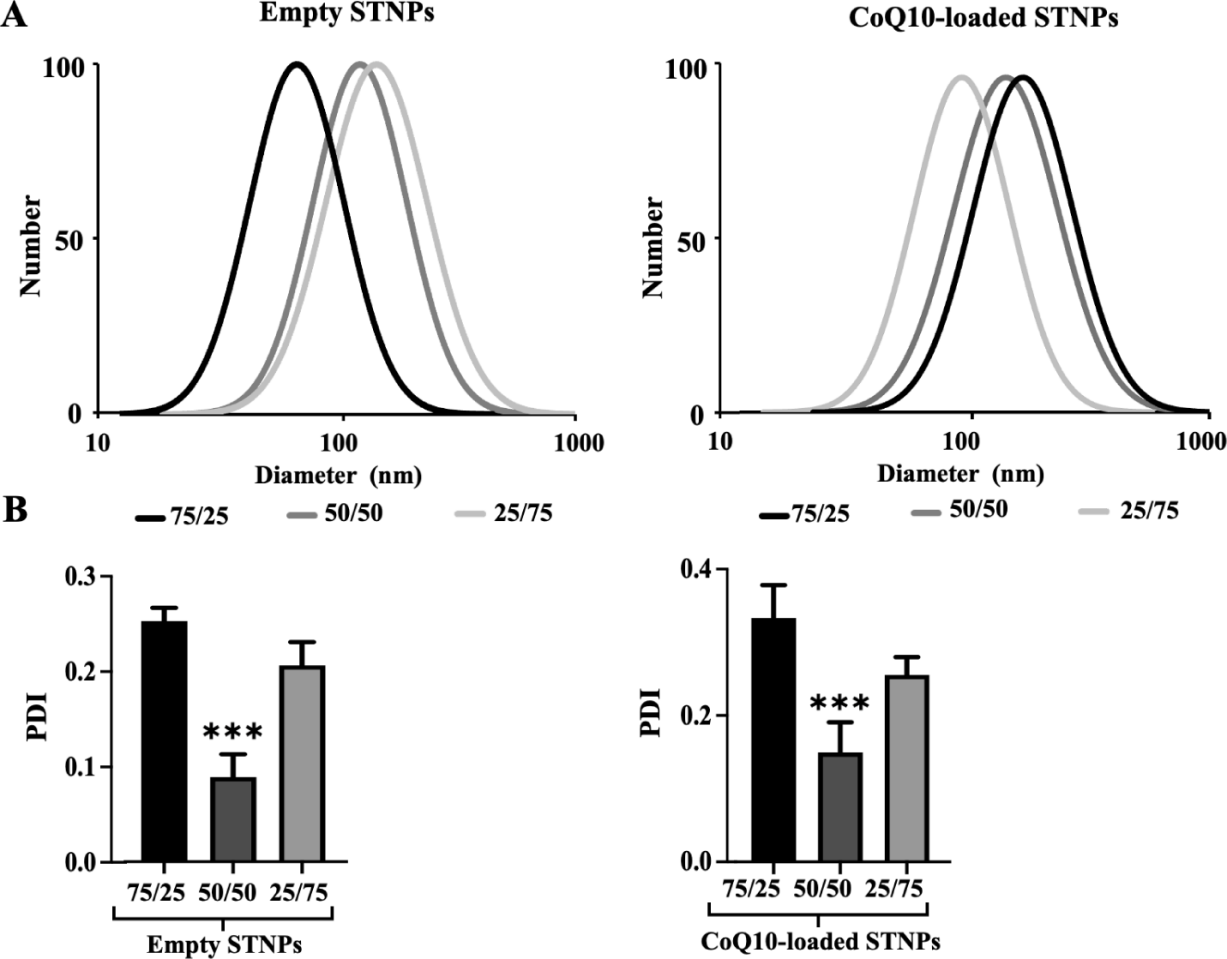
Particle size distribution (A) and PDI (B) of empty STNPs and CoQ10-loaded STNPs (mean ± SD, *n* = 3). STNP (50/50) was compared with STNP (75/25) and STNP (25/75). ***p < 0.001, statistically significant.

All of the empty NPs and drug-loaded NPs showed a similar zeta potential, ranging from −38.25 ± 0.827 to −43.50 ± 3.33 (Figure 5). The CoQ10 loading did not have a significant impact on the zeta potential of the NPs. The NPs’ negative zeta potential might be due to the high content of PS, which is negatively charged at physiological pH.^15,16^ The negative charge enhances the repulsion among NPs, preventing the formation of large aggregates, i.e., ensuring the stability of the NPs. The data were confirmed by NPs’ nanoscale size and low PDI (Figures 3 and 4).

**Figure 5:**
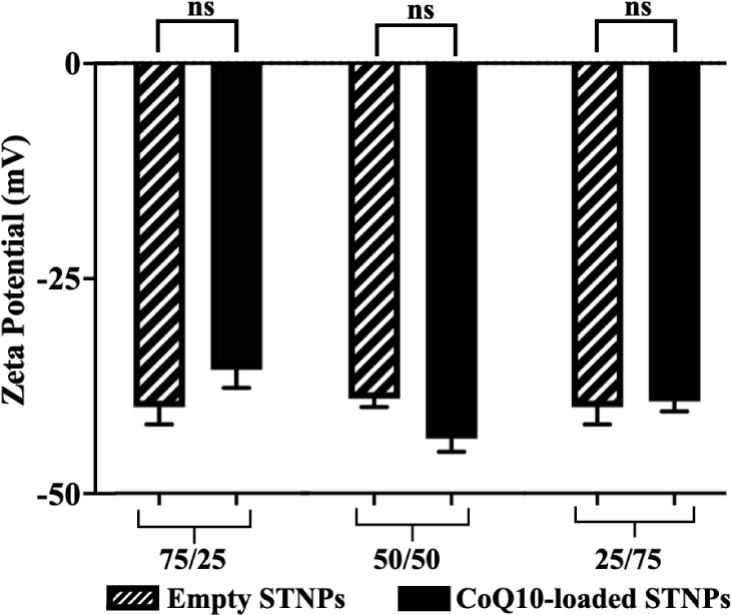
Zeta potential of empty NPs (STNPs) and CoQ10-loaded STNPs (mean ± SD, *n* = 3). ns, not statistically significant.

Here, the NPs/nanomedicines were prepared via self-assembly by using the conventional thin-film hydration method. We noticed that several NP parameters changed, including drug loading, particle size, zeta potential, PDI, etc., when the ratio of the NPs’ components was changed. This may be due to several factors related to polymer and lipid self-assembly, nanoparticle formation, and drug-lipid interactions.^63,64^ At different ratios, the required numbers of the “monomers” (i.e., polymers and lipids) to form an NP may not be the same, which could influence the NP parameters. After loading hydrophobic/lipophilic CoQ10, the drug—lipid/polymer interaction may influence nanoparticle packing, which might also change NPs’ particle size and zeta potential.

### 3.3 Sustained Drug Release

The *in vitro* drug release rate of the STNPs was determined by using a dialysis method. The drug release profile of the free CoQ10 and CoQ10-loaded STNPs, with or without MMPs, is shown in Figure 6. The free CoQ10 showed a rapid release, with more than 50% of the drug released within the initial 2 h due to the dissolution and diffusion behavior of free CoQ10. In contrast, the NPs demonstrated a sustained drug release pattern. To further analyze the data, the difference factor (f_1_) and similarity factor (f_2_) were calculated (Tables S1 and S2), as recommended by the Food and Drug Administration to assess the similarity of the dissolution profiles between formulations. The f1 values ranging from 0 to 15 and f2 values from 50 to 100 shows that similarity exists between two profiles.^45–200^ The f_1_ values for CoQ10 vs CoQ10-loaded STNPs (+MMPs and −MMPs) were 41.43 and 42.46, respectively, which are more than 15, indicating different drug release profiles. In contrast, the f_1_ value for CoQ10-loaded STNPs + MMPs vs CoQ10-loaded STNPs–MMPs was 7.97, which is less than 15, indicating no significant difference. The f_2_ values for CoQ10 vs CoQ10-loaded STNPs (+MMPs and −MMPs) were 33.76 and 33.57, respectively, which are less than 50, confirming dissimilarity, while the f_2_ value for CoQ10-loaded STNPs + MMPs vs CoQ10-loaded STNPs–MMPs was 76.97, which is more than 50, indicating similar drug release profiles.^45,46^

**Figure 6:**
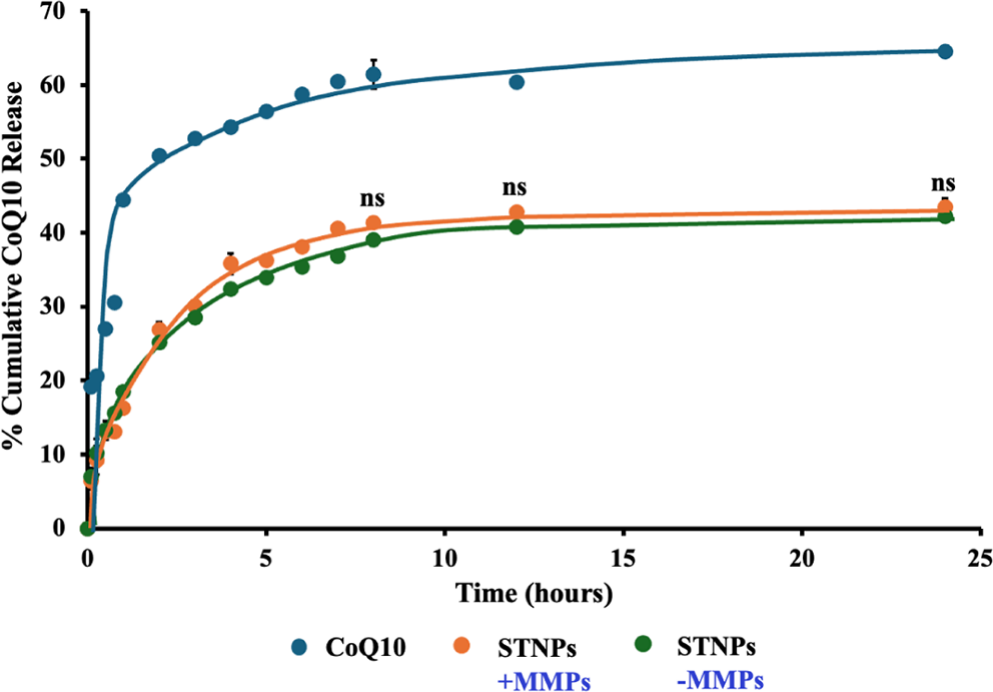
Drug release of CoQ10-loaded STNPs determined by a dialysis method under the “sink” condition (mean ± SD, *n* = 3). *ns*, not statistically significant.

The NPs’ sustained CoQ10 release profiles confirmed the core–shell-structured NPs’ excellent drug-loading capability and stability.^65,66^ The NPs, with or without MMP preincubation, showed almost the same drug release pattern, indicating that the influence of MMPs on drug release was negligible. This would ensure the stability of CoQ10-loaded NPs during drug delivery for macrophage-specific phagocytosis in inflamed tissues, minimizing undesirable side effects associated with “premature” drug release or leakage and providing more controlled and precise therapeutic effects in treating RA.

### 3.4 Stability of Nanoparticles at Different pHs and Temperatures

The stability of NPs was also evaluated through the measurement of particle sizes at different pH values and temperatures (Figure 7). In RA, along with disease progression, the pH in inflamed tissues decreases. In this experiment, the pH of the PBS was adjusted from 7.4 to 5.5 to represent the pH of normal tissues, inflamed tissues, and endo/lysosomes in RA.^67–71^ The NPs were also incubated at 4 °C (simulatingfor storage conditions), 25 °C (room temperature handling), or 37 °C (drug administration) for 72 h. Though particle sizes of the NPs were slightly changed under different conditions, they were in the proper nanoscale range, indicating NPs’ great colloidal stability during preparation, storage, and administration, most likely due to effective surface stabilization and steric repulsion.

**Figure 7:**
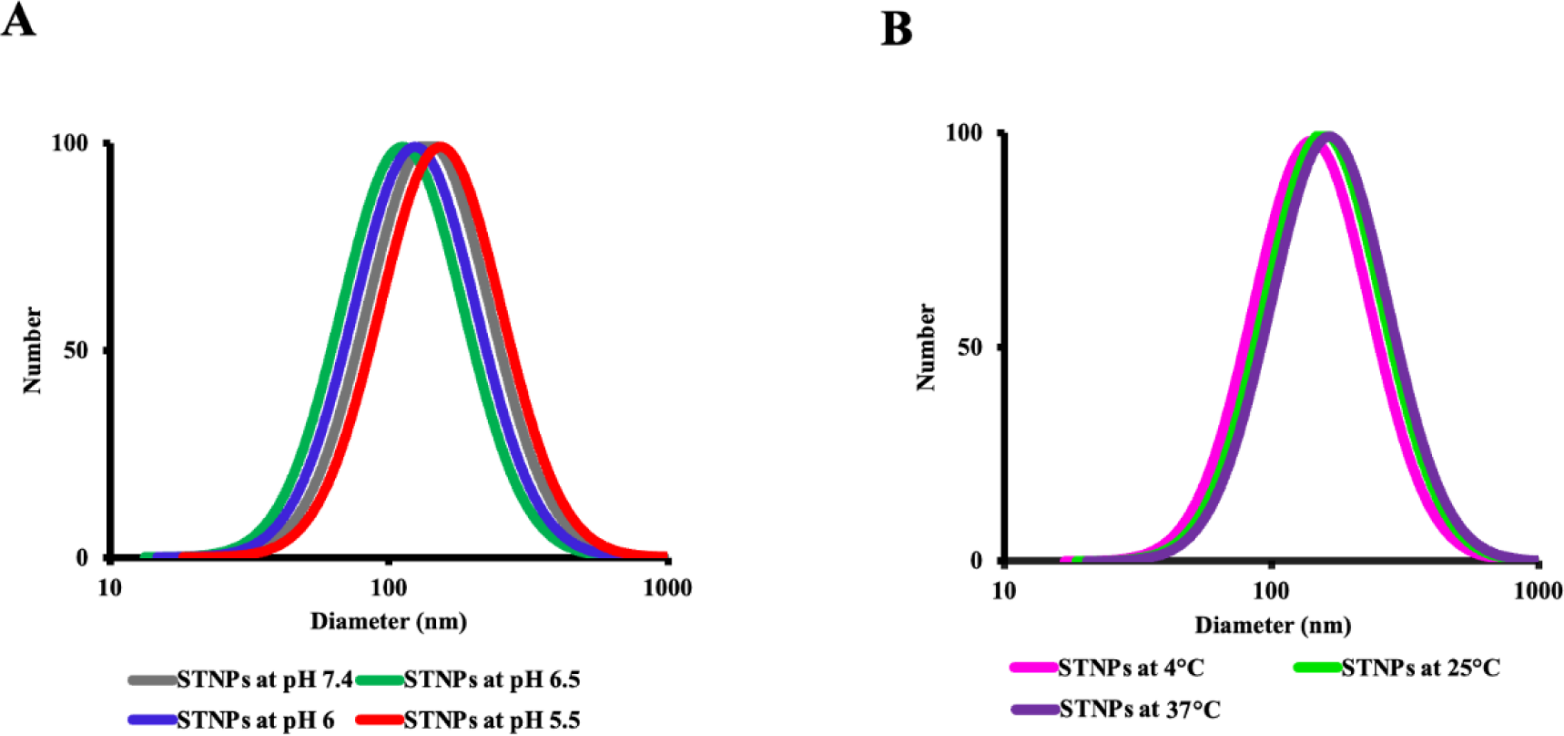
Particle size distribution of the STNPs at different pH values (A) and temperatures (B).

### 3.5 MMP-Dependent PS Exposure/“Externalization”

The MMP-sensitive PS exposure is critical to triggering effective phagocytosis in RA with MMP overexpression. To assess the MMP sensitivity and PS exposure, the STNPs were incubated with FITC-labeled annexin V, a commonly used probe for the detection of PS externalization in apoptotic cells. The binding affinity between the exposed PS and FITC-annexin V was examined by using flow cytometry. In this experiment, the PS NPs, having a strong binding affinity with FITC-annexin V, were used to represent the NPs with fully exposed PS. The PC NPs, showing low binding with FITC-annexin V, were used to represent the NPs with fully hidden PS. The STNPs were also tested in the presence or absence of MMPs.

The PEG-shielded (PS-hidden) STNPs had a low level of annexin V binding, comparable to the PC NPs, due to the PEG’s “stealth” effect. In contrast, the STNPs with the MMP preincubation showed a remarkably higher level of annexin V binding (Figure 8), suggesting that MMPs could effectively cleave PEG-pp-PE and expose the PS on the NPs. The results indicated that PS exposure on the surface of the NPs could be triggered and controlled by the MMPs.

**Figure 8:**
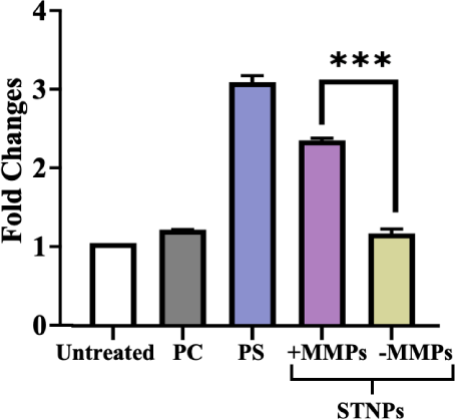
MMP-dependent PS exposure. The binding between FITC-Annexin V and NPs’ PS was analyzed by flow cytometry (mean ± SD, *n* = 3). STNPs were preincubated with MMPs at 37 °C for 4 h. ***p < 0.001, statistically significant.

### 3.6 MMP-Sensitive Cellular Uptake and Selectivity

To study the MMP sensitivity, the NPs were pretreated with the MMPs, followed by phagocytosis studies in mouse macrophages (RAW264.7). We found that the STNPs showed a significant increase in their uptake by macrophages in the presence of the MMPs compared to their counterparts in the absence of the MMPs (Figure 9). In contrast, both the MMP-sensitive, macrophage-nontargeting SNNPs (PEG-pp-PE/PC) and the nonsensitive, macrophage-targeting NTNPs (PEG-PE/PS) showed negligible cellular uptake regardless of whether they were preincubated with the MMPs or not.

**Figure 9:**
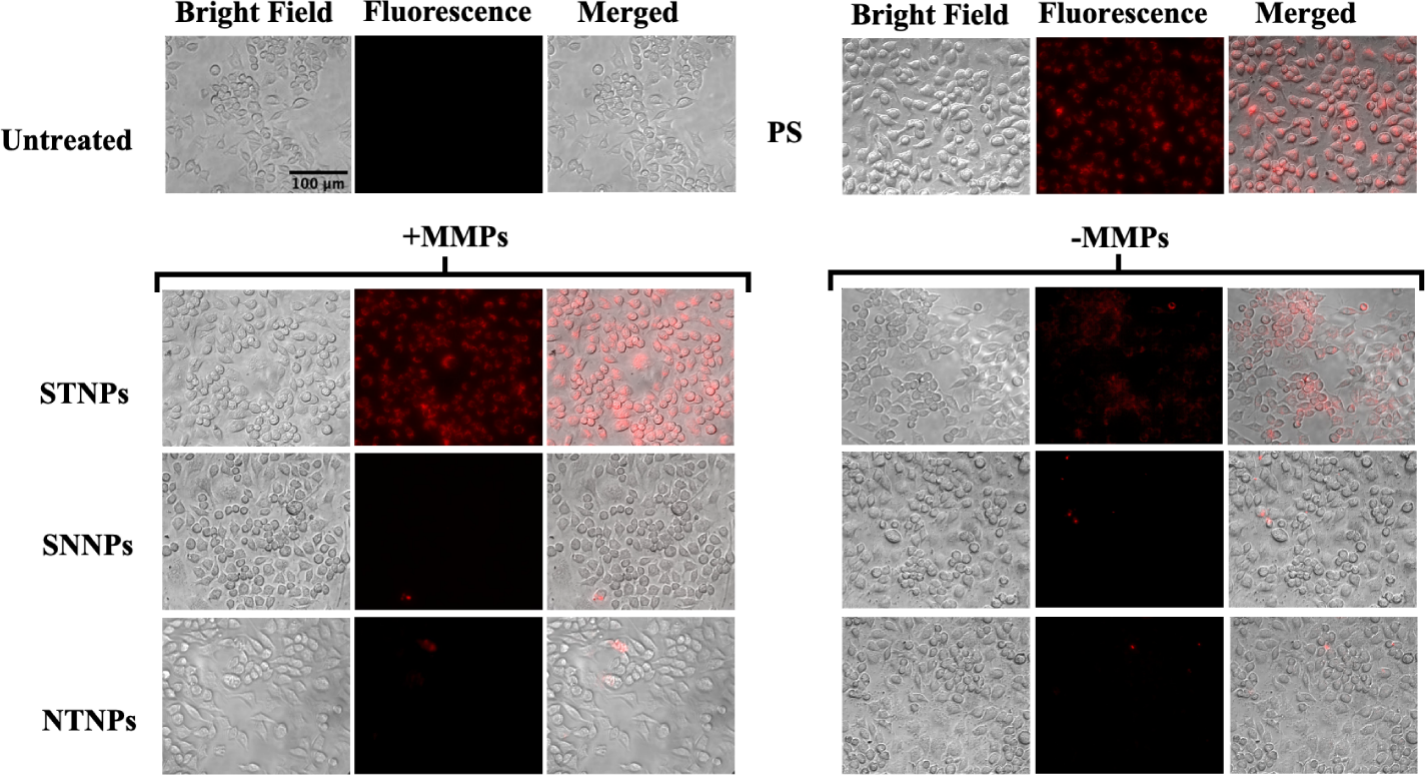
MMP2-sensitive cellular uptake of Rh-PE-loaded NPs in RAW 264.7 macrophages. **STNPs**, MMP- **S** ensitive macrophage **T** argeting PEG-pp-PE/PS **NPs** ; **SNNPs**, MMP-**S**ensitive macrophage **N** ontargeting PEG-pp-PE/PC **NPs** ; **NTNPs**, **N** onsensitive macrophage **T** argeting PEG-PE/PS **NPs** . The NPs were preincubated with or without MMPs for 4 h, followed by 1-h cell incubation. The treated cells were examined with a fluorescence microscope.

PEGylation is a well-known strategy to decrease NPs’ nonspecific interaction and increase NPs’ stability. Methoxy PEGs (mPEG) generally exhibit a slightly negative to neutral surface charge. The PEGylated NPs may be neutral if the PEG length and intensity are appropriate. However, even when charged, the PEGylated NPs could increase drugs’ specificity and decrease nonspecific uptake/biodistribution in many applications, including RA,^72^ cancer,^34,73,74^ etc. Our results suggested that the “stealth” PEG-PE (or intact PEG-pp-PE) could “mask” the PS and inhibit phagocytosis of PEGylated NPs. Upon MMP preincubation, the peptide linker (pp) of PEG-pp-PE was cleaved, leading to STNPs’ PEG deshielding and PS exposure. The PS-exposed NPs, acting as an “eat-me” signal, effectively triggered phagocytosis by macrophages. The data also suggested that when used alone, the MMP-sensitive PEG shielding/deshielding strategy (SNNPs) or the PS exposure for “eat-me” strategy (NTNPs) might not be effective for targeting macrophages in inflammation.

To evaluate the NPs’ cell selectivity, we compared the NP uptake in both macrophages (RAW264.7) and fibroblasts (NIH3T3) with or without MMP preincubation (Figure 10A). In NIH3T3 cells, most NPs, including PS NPs, had mild to low cellular uptake, and the STNPs showed no MMP responsiveness. The NPs with the exposed PS upon MMP preincubation failed to increase the cellular uptake, suggesting that PS was not a favorable factor for promoting NP internalization in NIH3T3 fibroblasts. However, under the same conditions, all NPs showed enhanced cellular uptake in RAW264.7 macrophages. Notably, the cellular uptake of MMP-preincubated STNPs in RAW264.7 cells was about 13-fold higher than that in NIH3T3 cells. Even without MMP preincubation, the STNPs still exhibited higher uptake in macrophages than in fibroblasts, possibly due to the endogenous MMPs produced by macrophages.^28^ Additionally, the uptake of the MMP-preincubated STNPs in macrophages was approximately 75-fold higher than the untreated group, while the uptake of the same formulations in fibroblasts was only approximately 6-fold higher than the untreated group (Figure 10B). All these data suggest that the STNPs are MMP-sensitive and macrophage-selective nanocarriers.

**Figure 10:**
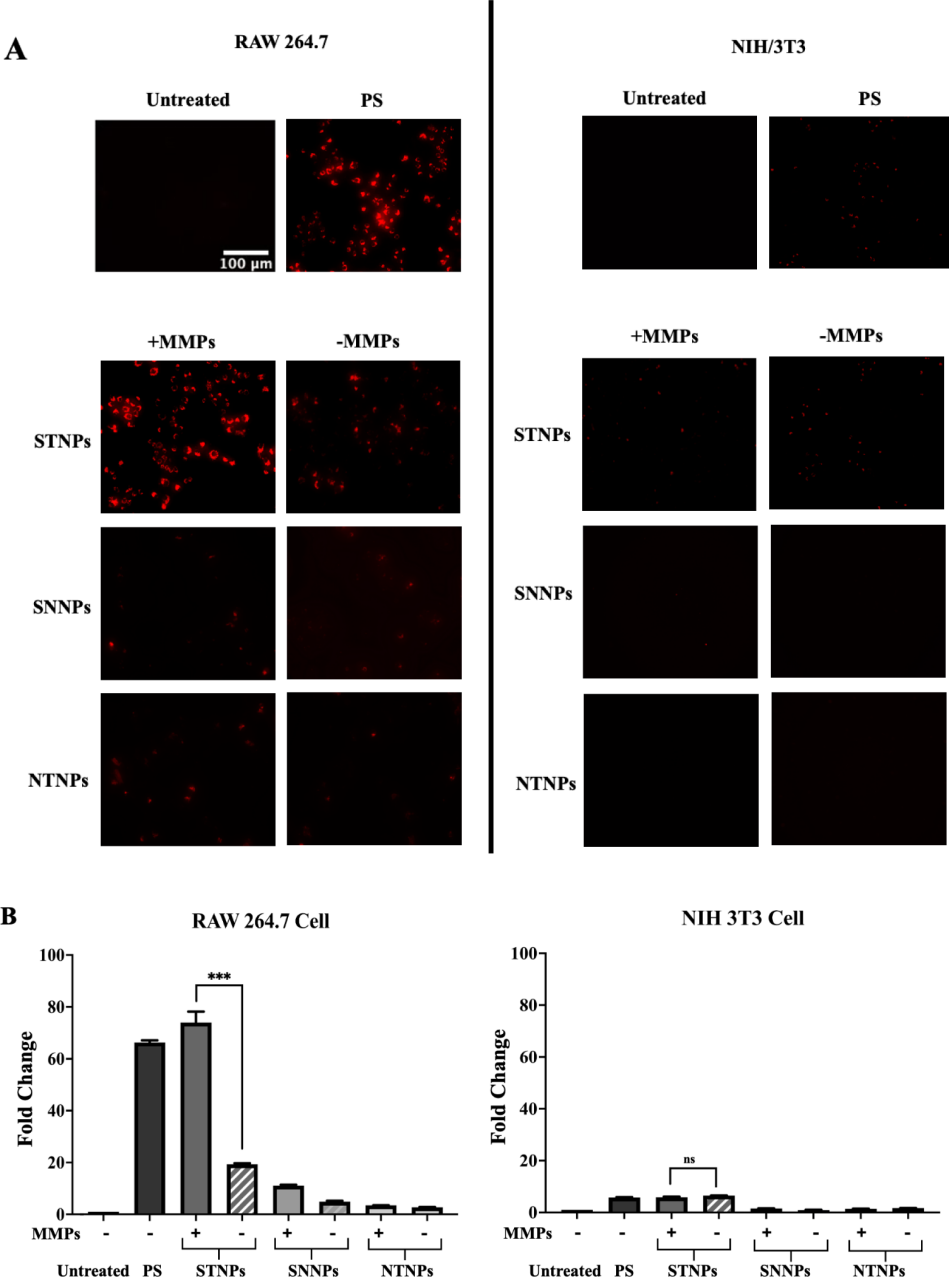
Cell selectivity of the Rh-PE-loaded NPs analyzed by fluorescence microscopy (A) and flow cytometry (B). **STNPs**, MMP- **S** ensitive macrophage **T** argeting PEG-pp-PE/PS **NPs** ; **SNNPs**, MMP-**S**ensitive macrophage **N** ontargeting PEG-pp-PE/PC **NPs** ; **NTNPs**, **N** onsensitive macrophage **T** argeting PEG-PE/PS **NPs** . The NPs were preincubated with or without MMPs for 4 h, followed by 1-h incubation with macrophages (RAW 264.7) and fibroblasts (NIH/3T3) (mean ± SD, *n* = 3). *ns*, not statistically significant; ***p < 0.001, statistically significant.

### 3.7 Cytotoxicity Assessment

The cytotoxicity of the STNPs loaded with CoQ10 was assessed in RAW 264.7 cells by the CellTiter-Blue Cell Viability Assay. The empty STNPs, free CoQ10, and CoQ10-loaded STNPs were tested at drug concentrations of 0.01–10 μg/mL. The typical human body contains 0.5–1.5 μg/mL of CoQ10, while disease-related conditions usually lower the CoQ10 concentration. Daily CoQ10 supplementation in the range of 100–300 mg can increase plasma CoQ10 levels to 2–5 μg/mL, whereas taking 1200 mg of CoQ10 per day may raise plasma concentrations to around 10 μg/mL.^75,76^ The tested CoQ10 concentrations in this study were clinically relevant.

After 48 h of treatment, no significant changes in cell viability were detected in any of the tested formulations compared to the untreated cells (Figure 11). The data suggested that both CoQ10 and CoQ10-loaded NPs were not toxic, even at high concentrations, in agreement with previous reports.^77,78^ This was not surprising because (i) CoQ10 is found in almost every cell in the human body and has been demonstrated to be safe as a dietary supplement,^79^ and (ii) the STNPs’ building materials are safe and have been widely used in drug formulations and delivery systems.^34,39,66^

**Figure 11:**
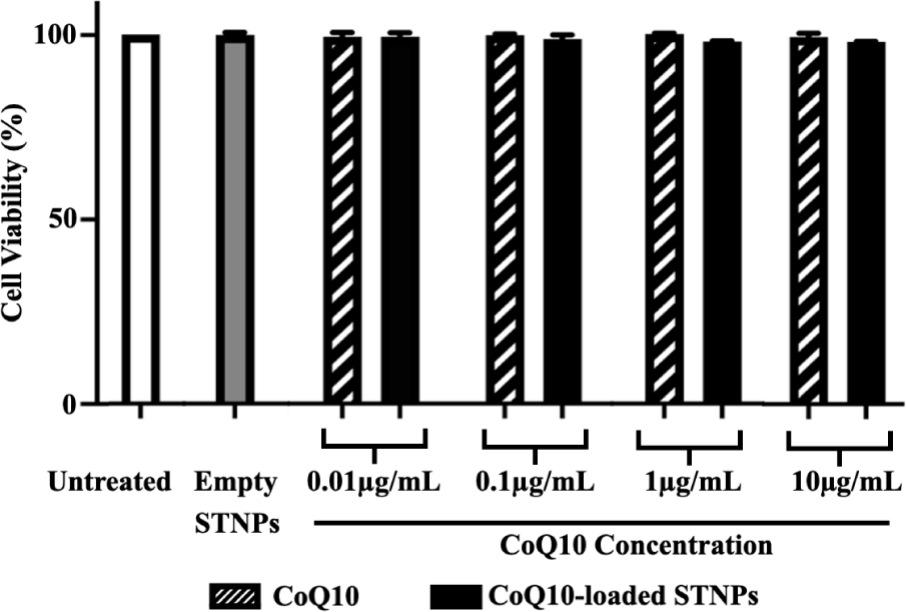
Cytotoxicity of free CoQ10, empty STNPs (100 μg/mL), and CoQ10-loaded STNPs. Cell incubation time: 48 h.

### 3.8 Inhibition of Inflammation by CoQ10-Loaded STNPs

In this work, we aimed to increase not only the bioavailability but also the specificity of CoQ10 by a novel nanoformulation to improve its anti-inflammatory activities and reduce its required doses. In this experiment, LPS+IFN-γ preincubation dramatically increased the production of inflammatory cytokines in macrophages, including TNF-α by approximately 7.5-fold, IL-6 by approximately 9-fold, and IL-1β by approximately 6.3-fold, as compared to untreated cells (Figure 12). In the LPS+IFN-γ-stimulated macrophages, free CoQ10 significantly decreased the levels of pro-inflammatory cytokines (TNF-α by approximately 4.6-fold, IL-6 by approximately 5.4-fold, and IL-1β by approximately 5-fold, compared to untreated cells), confirming its anti-inflammatory effects. We found that the CoQ10-loaded STNPs showed effects similar to those of free CoQ10 without MMP preincubation. However, after MMP preincubation, the CoQ10-loaded STNPs further reduced the TNF-α level to approximately 2.5-fold, the IL-6 level to approximately 3-fold, and the IL-1β level to approximately 3.1-fold, compared to untreated cells.

**Figure 12:**
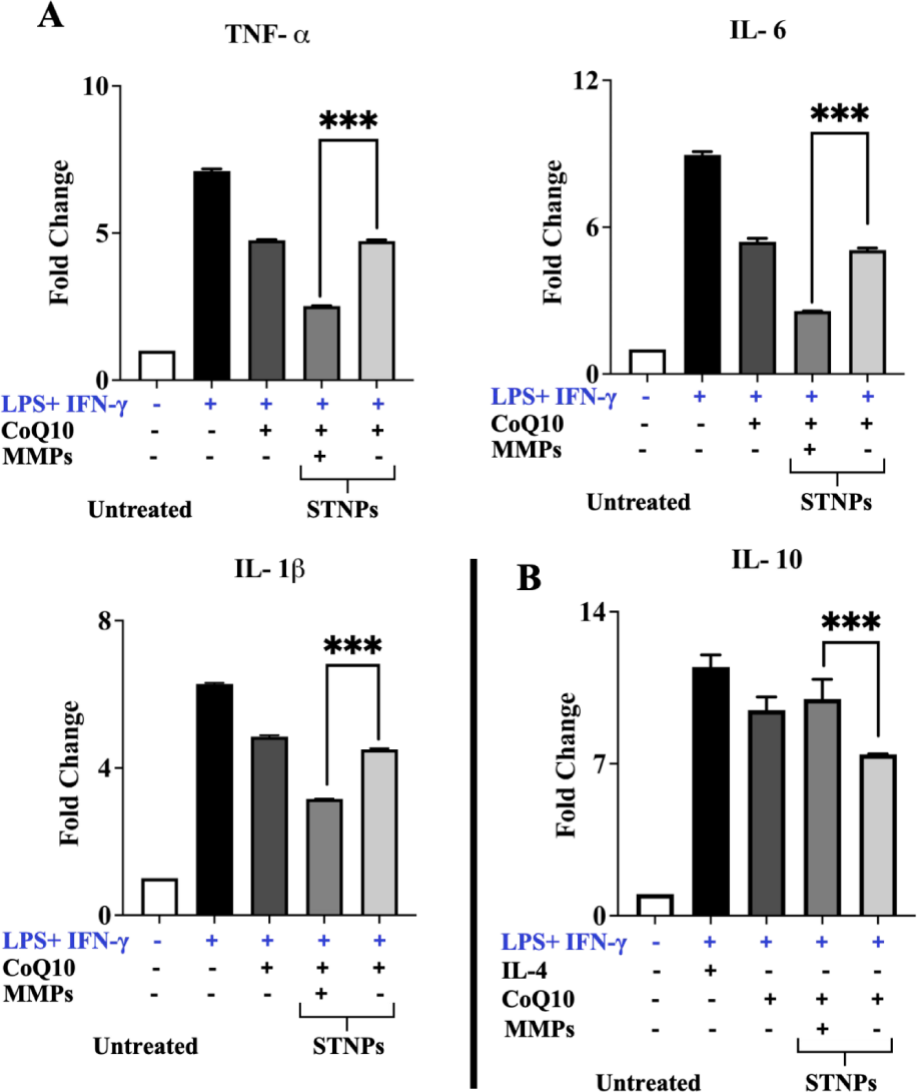
Inflammatory cytokines. The pro-inflammatory cytokines including TNF-α, IL-6, and IL-1β (A) and the anti-inflammatory cytokine, IL-10 (B) were determined by ELISA. RAW 264.7 cells were preincubated with LPS and IFN-γ for 24 h for activation, followed by various treatments for an additional 24 h. NPs’ MMP-preincubation time: 4 h. All results were shown as fold changes over untreated cells (mean ± SD, *n* = 3). ***p < 0.001, statistically significant.

To confirm the NPs’ effects on inflammation, the anti-inflammatory cytokine IL-10 was also measured in the LPS+IFN-γ-stimulated macrophages. Here, IL-4, which is well-known to promote IL-10 production, was used as a positive control. We found that free CoQ10 could increase the IL-10 level, which was similar to the IL-4 treatment.^80^ With MMP preincubation, the CoQ10-loaded STNPs significantly increased the IL-10 level compared to the NPs without MMP preincubation.

CoQ10 has been used to deal with inflammation in RA,^81,82^ but free CoQ10’s anti-inflammatory activities were moderate. All these results suggested that the STNPs could significantly improve the efficacy of CoQ10, and the CoQ10-loaded STNPs could effectively inhibit the production of pro-inflammatory cytokines while enhancing the production of anti-inflammatory cytokines in response to MMPs. Our strategy could improve both drug solubility and macrophage targetability/phagocytosis, thereby optimizing the therapeutic anti-inflammatory effects of CoQ10 in treating RA.

To confirm the effects of CoQ10 formulations on inflammation, the macrophage surface markers, including CD80, CD86, MHC II, and CD206, were analyzed by corresponding antibodies and flow cytometry.^83^ The overexpression of CD80, CD86, and MHC II indicates the macrophages’ pro-inflammatory polarization, while the overexpression of CD206 signifies that macrophages are polarized toward suppression of inflammation. In the experiment, free CoQ10 could only moderately downregulate CD80 and MHC II, but not CD86, in the stimulated macrophages (Figure 13). Loading CoQ10 into the STNPs increased CoQ10’s inhibitory effects compared to free CoQ10. The strongest inhibitory effects were observed with the MMP-pretreated CoQ10-loaded STNPs, which is in line with the cytokine production data (Figure 12). All CoQ10 formulations and IL-4 treatment substantially increased CD206 expression, with no significant differences observed among them, indicating the anti-inflammatory effects of these CoQ10-containing formulations. However, STNPs did not significantly increase CD206 expression upon MMP preincubation, which requires further investigation. Overall, the macrophage surface marker data confirmed that the STNPs could improve CoQ10’s anti-inflammatory effects, and these effects were MMP-sensitive.

**Figure 13:**
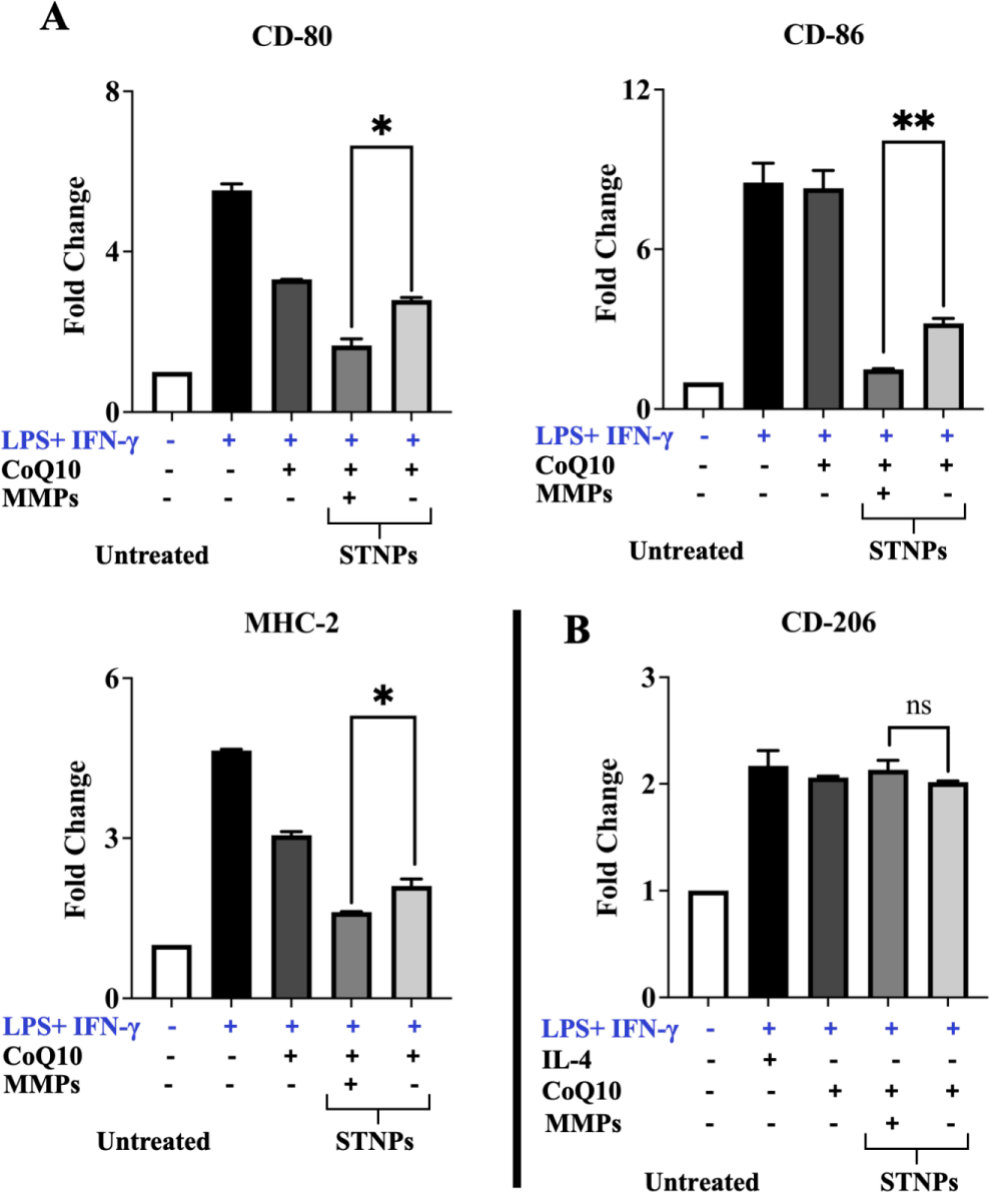
Macrophage surface markers. The pro-inflammatory markers, including CD80, CD86, and MHC II (A), and the anti-inflammatory marker CD206 (B), were analyzed by corresponding antibodies using flow cytometry. RAW 264.7 cells were preincubated with LPS and IFN-γ for 24 h for activation, followed by treatments for an additional 24 h. NPs’ MMP-preincubation time: 4 h. All results were shown as fold changes over untreated cells (mean ± SD, *n* = 3). *ns*, not statistically significant; *p < 0.05 and **p < 0.01, statistically significant.

To further evaluate the macrophage-selective anti-inflammatory effects of the CoQ10 nanomedicine, the macrophage/fibroblast cell coculture model was established. The expression of the pro-inflammatory cytokines TNF-α and IL-1β, as well as the surface markers CD80 and CD86, was analyzed. All CoQ10 treatments showed relatively lower therapeutic effects in the cell cocultures than in macrophages (Figure 14); however, the trends of the effects were similar. The free CoQ10 could only moderately inhibit inflammation. The MMP-pretreated CoQ10-loaded STNPs showed improved anti-inflammatory activity compared to the NPs without MMP pretreatment. The results suggested that the CoQ10-loaded STNPs could respond to the MMPs to selectively deliver CoQ10 to macrophages in the cell cocultures.

**Figure 14:**
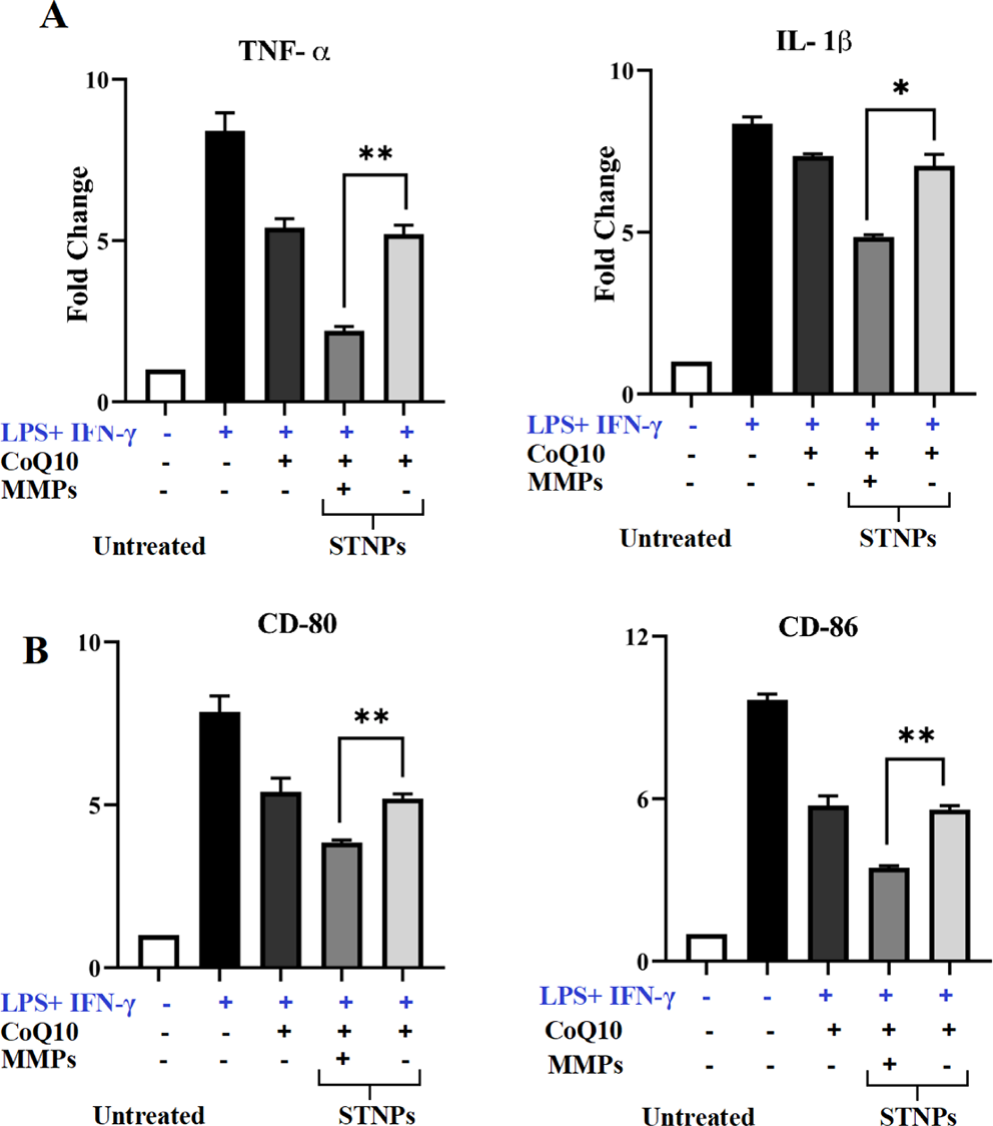
Cytokines and macrophage surface markers in the macrophage/fibroblast cell cocultures. The pro-inflammatory cytokines, including TNF-α and IL-1β (A), and cell surface markers, CD80 and CD86 (B), were analyzed by ELISA and flow cytometry. The cocultures were preincubated with LPS and IFN-γ for 24 h to mimic RA inflammation, followed by treatments for an additional 24 h. NPs’ MMP preincubation time: 4 h. All results were presented as fold changes over untreated cells (mean ± SD, *n* = 3). *ns*, not statistically significant; *p < 0.05 and **p < 0.01, statistically significant.

The impact of CoQ10-loaded STNPs on macrophage activation was also examined by cells’ morphology. After 24-h activation by LPS and IFN-γ, the RAW 264.7 cells displayed a significant extension of pseudopodia, which are temporary, arm-like projections of the cell membrane that play a crucial role in phagocytosis.^84^ The number and length of pseudopodia extensions notably increased with the duration of activation. Upon treatment with CoQ10-loaded STNPs, the pseudopodial extensions of the activated macrophages were inhibited. The effects of CoQ10-loaded STNPs were dose-dependent, and at a CoQ10 dose of 2.5 μg/mL, the LPS+IFN-γ-induced pseudopodia extensions had almost completely disappeared in macrophages, resembling those of untreated normal cells (without activation) (Figure 15). In contrast, macrophages treated with 2.5 μg/mL free CoQ10 largely retained intact pseudopodial extensions, mirroring the morphology of the activated macrophages. The improved activity of CoQ10-loaded STNPs was mainly attributed to the increased drug solubility and enhanced phagocytosis. The data were consistent with the macrophages’ cytokine production and surface marker expression.

**Figure 15:**
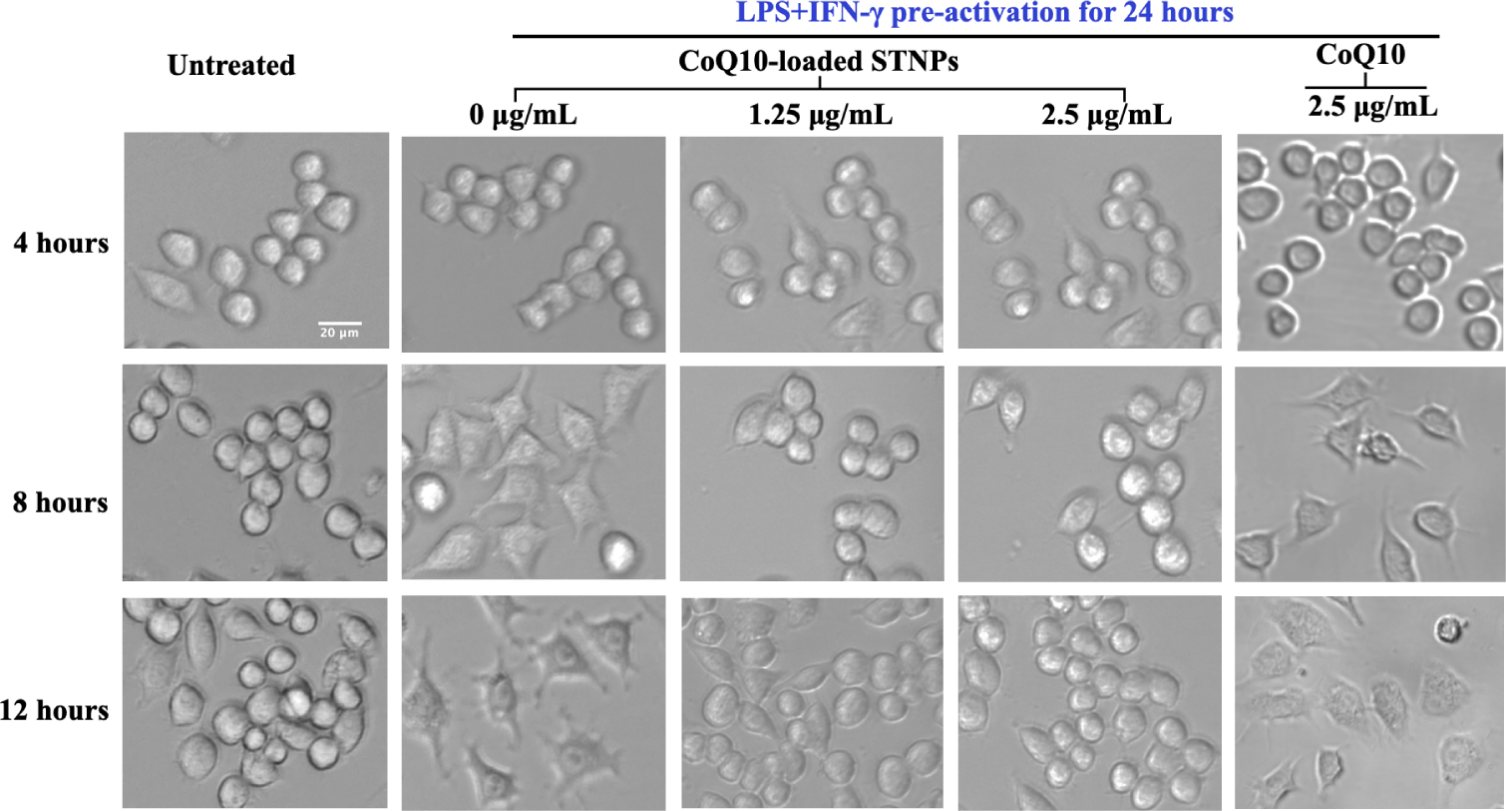
Macrophage morphological changes. RAW 264.7 cells were preactivated with LPS + IFN-γ for 24 h, followed by incubation with CoQ10-loaded STNPs at CoQ10 doses of 0, 1.25, or 2.5 μg/mL for up to 12 h.

Macrophages serve as the front line of the immune defense and are integral to numerous physiological and pathological processes. Macrophages can identify invaders like bacteria and viruses, dead cells, and debris, as well as foreign substances such as biomaterials and NPs. Specific molecular patterns act as signals for macrophages to engulf and clear out pathogens. In the inflamed synovium, in addition to immune cells (macrophages and T cells), fibroblasts (fibroblast-like synoviocytes) are a major cell type.^85^ Elevated levels of pro-inflammatory cytokines and enzymes, including MMPs such as MMP2 and MMP9,^6^ have also been found in inflamed tissues. Although these MMPs may also exist in normal tissues and organs such as the liver, spleen, etc., their expression and activity are usually low compared to the MMPs in inflamed joints in RA.^86–88^ Additionally, the Enhanced Permeability and Retention (EPR) effect is prominent in inflamed tissues,^13^ which facilitates the passive accumulation of NPs in the joints of RA patients.^89^ Accordingly, several MMP2/9-responsive NP systems, such as liposomes and micelles, have been developed for treating inflammatory diseases such RA.^38,89^ However, these drug delivery systems were not macrophage-specific. In our design, the NPs’ MMP-sensitive PS exposure worked as an “eat me” signal, leading to phagocytosis by macrophages rather than nonphagocytic cells, such as fibroblasts. The STNPs’ ability to target inflammation-associated macrophages can increase the precision of drug delivery, which may decrease CoQ10 doses, enhance CoQ10’s therapeutic effects, and minimize off-target adverse effects for effective RA treatment.^90,91^

## 4 Conclusion

In this study, we prepared a smart nanomedicine for the targeted delivery of CoQ10 to inflammation-associated macrophages. The prepared nanomedicine demonstrated high CoQ10 loading, excellent physicochemical properties, and sustained drug release. The nanomedicine was safe and showed efficient, MMP-dependent cellular uptake in macrophages rather than in nonphagocytic cells. In macrophages activated by LPS and IFN-γ, the CoQ10 nanomedicine significantly reduced the levels of pro-inflammatory cytokines and macrophage activation markers while elevating the levels of anti-inflammatory cytokines and macrophage markers, leading to effective inhibition of inflammation. Our results suggest that the developed MMP-sensitive macrophage-targeted CoQ10 nanomedicine has great potential for RA treatment.

## Supplementary Material



## Notes

Difference factor (f1) and similarity factor (f2) (PDF)
