# Supplementary material for: MMP-Sensitive Macrophage-Targeted Coenzyme Q10 Nanomedicine for Rheumatoid Arthritis Treatment
Source: Mol Pharm. 2025 Aug 5;22(9):5638–51. doi: 10.1021/acs.molpharmaceut.5c00742 (PMC12406254; doi:10.1021/acs.molpharmaceut.5c00742)
Supplement: Supplementary file 1 [file mp5c00742_si_001.pdf]

Supporting information for

MMP-sensitive Macrophage-targeted Coenzyme Q10 Nanomedicine for Rheumatoid Arthritis Treatment

Aasma Akram[1,2,] ↑, Nishat Ara[1], ↑, Prapanna Bhattarai[1], Muhammad Irfan[2],*, Lin Zhu[1],*

1 Department of Pharmaceutical Sciences, Irma Lerma Rangel College of Pharmacy, Texas A&M University, College Station, Texas 77843, United States.

2 Department of Pharmaceutics, Faculty of Pharmaceutical sciences, Government College University, Faisalabad 38000, Pakistan.

↑These authors contributed equally to this work.

*Corresponding authors:

E-mail address: lzhu@tamu.edu (Lin Zhu)

E-mail address: drmirfan@gcuf.edu.pk (Muhammad Irfan)

Difference factor (f1) and similarity factor (f_2) were applied to compare dissolution profiles of drug and formulations using DDSolver software [1]. The f_1 values ranging from 0 to 15 and f_2 values from 50 to 100 shows that similarity exists between two profiles [2, 3].

Table S1: Difference factor (f_1) of CoQ10 and CoQ10-loaded STNPs.

**Formulations**	STNPs +MMPs	STNPs –MMPs
CoQ10	41.43	42.46
STNPs +MMPs	-	7.97

Table S2: Similarity factor (f_2) of CoQ10 and CoQ10-loaded STNPs.

**Formulations**	STNPs +MMPs	STNPs –MMPs
CoQ10	33.76	33.57
STNPs +MMPs	-	76.97

REFERENCES:

(1) Zhang, Y.; Huo, M.; Zhou, J.; Zou, A.; Li, W.; Yao, C.; Xie, S. DDSolver: an add-in program for modeling and comparison of drug dissolution profiles. *AAPS J* **2010**, *12* (3), 263-271. DOI: 10.1208/s12248-010-9185-1 From NLM Medline.
(2) Boddeda, B.; Kumari, P.; Chowdary, K. Formulation and evaluation of glipizide sustained release tablets. *International Journal of Pharmaceutics* **2012**, *3* (1), 44-48.
(3) Zhai, S.; Mathew, T.; Huang, Y. Comparison of drug dissolution profiles: a proposal based on tolerance limits. *Statistics in Medicine* **2016**, *35* (29), 5464-5476.
